# Isolation and Identification of Coliform Bacteria and Multidrug-Resistant *Escherichia coli* from Water Intended for Drug Compounding in Community Pharmacies in Jordan

**DOI:** 10.3390/healthcare11030299

**Published:** 2023-01-18

**Authors:** Mohammad K. Abu-Sini, Rafeef A. Maharmah, Dina H. Abulebdah, Mohammad N. S. Al-Sabi

**Affiliations:** 1 Department of Pharmacy, Faculty of Pharmacy, Al-Zaytoonah University of Jordan, P.O. Box 130, Amman 11733, Jordan; 2 Department of Microbiology, College of Veterinary Medicine, King Faisal University, P.O. Box 400, Al-Ahsa 31982, Saudi Arabia

**Keywords:** water, drug reconstitution, community pharmacies, contamination, coliform bacteria, multidrug resistance

## Abstract

(1) Background: Water is necessary for the preparation of some medicines found in pharmacies where the local water source does not meet the required purity. This study aimed to investigate the presence of coliform contamination in water used for drug reconstitution in community pharmacies in Jordan. (2) Methods: Two water samples from 50 randomly selected community pharmacies representing all Jordanian governorates were filtered and then cultured in plate count agars to determine total microbial count, and in m-Endo Agar Les and Eosin Methylene Blue (EMB) agar to cultivate *Escherichia coli* (*E. coli*). The presence of *E. coli* was further characterized with gram stains, biochemical tests, and Polymerase chain reaction (PCR). Antibiotic susceptibility of isolated *E. coli* was tested against a variety of standard antibiotics. (3) Results: Community pharmacies used droppers filled with water from coolers (62%), bottled water (20%), boiled tap water (16%) and tap water (2%). The majority of the sampled water contained coliform bacteria (88%), and *E. coli* was isolated from 26% of all samples. Statistical analysis showed no significant difference in the percentage of contaminated water samples based on its source location. Nonetheless, the results showed a tendency for higher proportions of contamination in droppers filled from boiled tap water (37.5%; SE: 17.1), followed by water from water coolers (25.8%; SE: 7.9), and then from bottled water (20%; SE: 12.7). All of the isolated *E. coli* were sensitive to gentamycin, ciprofloxacin and levofloxacin. The susceptibility of the isolates to ceftazidime, doxycycline, tetracycline, azithromycin and amoxicillin/clavulanic acid were 92%, 61%, 46%, 23% and 15%, respectively. (4) Conclusions: This study confirms the widespread presence of multidrug-resistant bacteria in water intended for reconstituting drugs in local pharmacies. These findings expose an alarming situation that needs special attention by the acting pharmacists and competent authorities. Higher levels of personal hygiene in the pharmacies coupled with regular inspection of water quality may reduce the risk of microbial contamination in compounded products, especially multidrug-resistant strains of *E. coli* and other index microorganisms.

## 1. Introduction

Water is considered a carrier of faecal-borne disease. The consumption of such contaminated water can lead to infection with many bacterial, viral and protozoal diseases [1]. However, water is one of the primary requirements for most pharmaceutical endevors as a raw material, ingredient and solvent in the processing, formulation and manufacturing of pharmaceutical products, active pharmaceutical ingredients (APIs) and intermediates [2,3]. Accordingly, water used in pharmaceutical preparations must be of higher purity than that usually available in domestic water sources [4]. Therefore, water used to produce pharmaceutical products must fulfil quality requirements as dictated in published standards. According to those standards, pharmacies and pharmaceutical companies set up special systems for water purification, which constitute an important part of the infrastructure of their institutions [5,6,7].

Bacteria, viruses and protozoa are microbes that may be present in water as pollutants. Coliform bacteria are microbes that emerge from the intestinal tracts of warm-blooded animals and exist in soil and vegetation. The existence of coliform bacteria in water indicates the possibility that disease-causing bacteria are also present in the water [8]. Hence, applying bioburden testing in water samples establishes the number of existing microorganisms, ensuring that bacterial loads do not exceed mandated pharmacopeia indices. Microbial testing of water includes the estimation of the number of specific index bacteria present in a given quality of water, which includes *Escherichia coli* (*E. coli*), *Staphylococcus aureus*, *Pseudomonas aeruginosa* and *Aspergillus niger* [9].

*E. coli* belongs to the *Enterobacteriaceae* family and is described as a facultative anaerobic, gram-negative, non-spore-forming, rod-shaped bacterium that contains the enzyme β-glucuronidase [10]. *E. coli* is an extremely versatile bacteria with over 250 serotypes, ranging from innocuous gut commensals to intra- or extra-intestinal pathogens that can colonize common medical devices, and is considered the primary cause of recurrent urogenital infections [11,12,13]. *E. coli* is commonly found in faeces, and its presence in pharmaceutical preparations is considered a direct indicator of faecal contamination in those products. Products contaminated with *E. coli* are discarded due to the possible presence of enteric pathogens, which include some pathogenic strains of *E. coli*, such as 0157: H7 [14].

Globally, antimicrobial resistance is a growing concern that poses a threat to public health. Antibiotic resistance is expected to cause increased morbidity and mortality rates that may reach as high as ten million extra deaths by 2050, in addition to increases in healthcare expenditure [15,16]. One of the major potential reservoirs for antimicrobial resistance genes has been identified in the commensal *E. coli* of humans and animals [17]. Moreover, these genes can transfer to *E. coli* or to other bacteria found in people, animals, and in the environment [18], which complicates the effort to control the spread of antibiotic resistance.

To the best of our knowledge, no work has been published on the occurrence of *E. coli* as an indicator of coliform contamination in water used for the reconstitution of drugs in Jordanian community pharmacies. Therefore, this work was conducted to fill this knowledge gap.

## 2. Materials and Methods

### 2.1. Sampling Plan

This microbial study was conducted in the microbiology laboratory at the Faculty of Pharmacy at Al-Zaytoonah University of Jordan in July 2020. Sampling of water used for drug compounding was conducted on water samples collected from 50 randomly selected community pharmacies representing all governorates of Jordan. In Jordan, water used for drug compounding in the community pharmacies is directly taken from water droppers, with the source of that water being either bottled water, tap water, boiled tap water, or water from coolers. Therefore, water was sampled from the droppers that are continuously refilled without prior or subsequent sterilization or treatment.

### 2.2. Collection of Water Samples

Two water samples were collected aseptically from the sampled pharmacies in a sterile bottle with a cap, each of 100 mL capacity. The bottles were labelled with a code number corresponding to the pharmacy and the date of collection and were stored immediately in an icebox for transportation. The volume of each water sample was around 100 mL. Samples were collected between 10 a.m. and 2 p.m. [19] and were immediately transported to the microbiology laboratory. Microbiological examination was started promptly to avoid unpredictable changes (preferably within 2 hrs of arrival).

### 2.3. Bacterial Propagation and Identification

The sampled water was tested for microbial contamination using the total plate count (TPC) and total coliform count (TCC) methods. Four different types of media were used for the propagation, isolation and detection of *Enterobacteriaceae*. These media included: plate count agar medium (Biolab, Budapest, Hungary), for enumeration of total microorganisms in water samples, m-Endo Agar Les medium (Liofilchem, Italy) for the detection and enumeration of coliforms in water samples, and MacConkey agar and Eosin Methylene Blue medium (Biolab, Budapest, Hungary) for the isolation, enumeration and differentiation of *E. coli*. The above media were prepared according to manufacturers’ instructions. Sterility of the four prepared media types was achieved by autoclaving at 121 °C for 15 min.

### 2.4. Detection and Enumeration of Total Viable Microorganisms, Total Coliforms and E. coli by Membrane Filtration

The recommended techniques used to determine the faecal contamination of water by *E. coli* are the multiple tube fermentation method and the membrane filtration method [20], the latter of which was implemented in this study. For all samples, two volumes of about 100 mL were filtered through 0.45 μm pore-sized nylon membrane filters (Agela Technologies, China) using a sterile filtration unit (Thermo Scientific Nalgene Filtration Products, Nalgene, Mexico) and vacuum pump (Vp 280′2831). The membranes were aseptically removed using sterile forceps, rotated upside down and placed on plates of plate count agar and m-Endo Agar Les, ensuring that no air bubbles were trapped. The plates were then incubated at 35 ± 0.5 °C for 22 to 24 h. Colonies of *E. coli* exhibited a distinctive pink-to-dark red colour with a metallic green sheen in the EMB agar.

Coliform density was reported as the number of colonies per 100 mL of sample. Samples of sterile distilled water were used as negative controls. One strain of *E. coli* (ATCC 8739) was mixed with 100 mL sterile distilled water, filtered as above, and used to produce 20–80 coliform colonies per filter. This sample was used as a positive control to enumerate coliform density according to the following equation:

$$\mathrm{Coliform}\;\mathrm{colonies}\;\mathrm{per}\;100\;\mathrm{mL}\;=\frac{\mathrm{coliform}\;\mathrm{colonies}\;\mathrm{counted}}{\mathrm{mL}\;\mathrm{of}\;\mathrm{original}\;\mathrm{sample}\;\mathrm{filtered}}\times 100$$

If growth covered the entire filtration area of the membrane, or a portion of it without discrete colonies, the results were then reported as “Confluent Growth With or Without Coliforms.” If the total number of colonies (coliforms plus non-coliforms) exceeded 200 per membrane, or the colonies were too indistinct for accurate counting, the results were reported as “Too Numerous to Count (TNTC)” [20].

### 2.5. Identification of E. coli

*E. coli* isolates were identified based on colonial morphology and with gram staining. Under light microscopy, *E. coli* cells are typically gram negative and are short and rod-like in appearance. Conventional biochemical tests, which included catalase and oxidase tests, were used for further characterization of the bacteria, according to methods described elsewhere [20]. Further identification of the isolates was performed using the HB010 Hi *E. coli*™ Identification Kit (HiMedia Laboratories, India), which is a standardized colorimetric identification system that uses eight conventional biochemical tests and four carbohydrate utilization tests.

### 2.6. Identification of E. coli by PCR

#### 2.6.1. DNA Extraction

Extraction of DNA from propagated bacterial cells was performed using the G-spin Total DNA Extraction Kit according to manufacturer’s instructions (iNtRON Biotechnology, Seoul, Korea). Briefly, a pure single colony of *E. coli* was transferred from a nutrient agar plate (Biolab, Budapest, Hungary) into 5 mL nutrient broth media (Biolab, Budapest, Hungary) and then incubated overnight at 37 ℃ until it obtained an OD600 value of 0.8~1.0 on a spectrophotometer (UV-M51, BelEngineering, Monza, Italy). Then, 1–2 mL of the bacteria suspension was transferred to a 2 mL tube and centrifuged for 1 min at 13,000 rpm. Next, 200 μL Buffer CL, 20 μL Proteinase K and 5 μL RNase A solution were added to the sample tube and mixed using a vortex. The lysate was then incubated at 56 ℃ for 10–30 min in a water bath. Then, 200 μL of Buffer BL was added to the sample tube, mixed thoroughly, and incubated at 70 °C for 5 min. Finally, the suspension was centrifuged at 13,000 rpm for 5 min and the supernatant was collected and used as a DNA template for PCR.

#### 2.6.2. PCR for Isolates Identification

PCR was performed to confirm the identity of the propagated isolates by detecting the β-galactosidase gene using the *E. coli*-specific lacZ3 primers (Table 1), as previously described [21]. All PCR reactions were done using a MultiGene Conventional PCR machine (Labnet, Edison, New Jersey, USA). The gene was amplified using 5 μL of PCR Master Mix 5× (FIREPol^®^ Master Mix Ready to Load, Solis BioDyne, Estonia). The volume was made up to 25 μL using nuclease free water (Integrated DNA Technologies, Coralville, IA, USA). Amplification was done by initial denaturation at 95 °C for 3 min, followed by denaturation at 95 °C for 30 s; annealing temperature of primers was 58 °C for 30 s and extension at 72 °C for 1 min. The final extension was conducted at 72 °C for 10 min and the total reaction was performed for 37 cycles. The amplified PCR products were analysed by electrophoresis in 1.5% agarose gel at 100 v (NanoPAC Power supply, Cleaver Scientific, Rugby, UK) for 45 min, stained with ethidium bromide, and finally, visualized with UV Transilluminator.

### 2.7. Antibiotic Susceptibility Test

To measure the antimicrobial drug susceptibility of all *E. coli* isolates, the Kirby–Bauer disk diffusion method was used according to the Clinical and Laboratory Standards Institute guidelines [22]. Susceptibility patterns of the isolates were determined against doxycycline (DO, 30 mcg), ceftazidime (CAZ, 30 mcg), gentamicin (CN, 10 mcg), ciprofloxacin (CIP5, 5 mcg), azithromycin (AZM, 15 mcg), amoxicillin/clavulanic acid (AMC, 30 mcg), levofloxacin (LVX5, 5 mcg) and tetracycline (TE, 30 mcg). All standard antibiotic discs were obtained from Oxoid (Basingstoke, Hampshire, UK). The results of antimicrobial testing were recorded as sensitive, intermediate sensitive and resistant according to zone diameter interpretative standards [22]. *E. coli* ATCC 8739 strain was included as a positive control.

### 2.8. Statistics

Data were transferred to Microsoft Excel^TM^ to generate statistical summaries. Statistically significant difference between the sources of contaminated water samples was based on *p*-values of >0.05, inferred from the Z-score for the differences between the means or proportions of infections.

## 3. Results

### 3.1. Detection and Enumeration of Total Coliforms and E. coli

**Figure 1:**
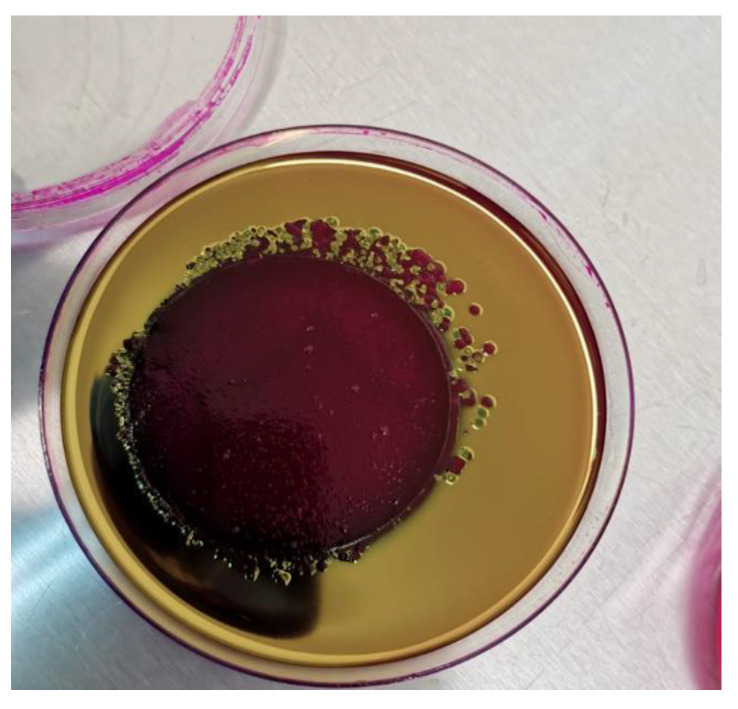
Colonies of *Escherichia coli* grown on m-Endo Agar LES, producing a dark-red metallic sheen.

The bacteria were cultured in plate count agar to determine total microbial count; m-Endo Agar LES was used, which encourages *E. coli* growth as red-coloured colonies with a metallic green sheen (Figure 1).

Coliform contamination was detected in 44 of the 50 water samples (88%) collected from community pharmacies in Jordan (Table 2). Only 12% (6 of 50) of water samples were free of coliform microbial contamination. Total microbial colonies were too numerous to count (TNTC) in most of the sampled water (58%; 29 of 50), while 30% of the samples (15 of 50) had countable total microbial colonies with a range of 5–30 CFU/100 mL. The presence of *E. coli* was confirmed in 13 samples of the collected 50 samples (26%; 95% confidence interval: 14.6–40.4%). The total coliform count in the water samples contaminated with *E. coli* had a range of 1–10 CFU/100 mL, except for one sample that produced TNTC.

The majority of the community pharmacies filled the droppers with water from water coolers (n = 31), followed by bottled water (n = 10), boiled tap water (n = 8), and then tap water (n = 1, Table 3). With the exception of the latter group, statistical analysis showed no significant difference in the proportion of contaminated water samples based on its source. Nonetheless, the results showed a tendency for higher proportions of contamination in droppers filled from boiled tap water (37.5%; SE: 17.1), followed by droppers filled from cooler water (25.8%; SE: 7.9), then droppers filled from bottled water (20%; SE: 12.7).

### 3.2. Identification of E. coli

**Figure 2:**
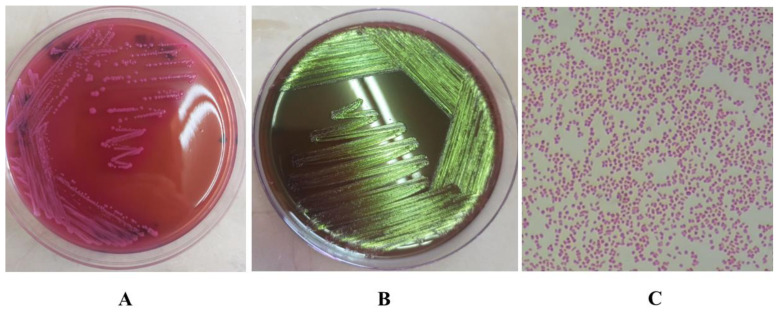
(**A**). The growth of *Escherichia coli* on MacConkey agar show colonies that are dry, donut shaped and dark pink in colour, surrounded with dark pink area of precipitated bile salts. (**B**). Growth of *E. coli* on EMB agar shows the characteristic green metallic sheen. (**C**). Morphology of *E. coli* cells by microscopy after gram staining, showing gram negative, short rods.

In this study, 13 isolates of coliform bacteria were identified as *E. coli* (26%). The identification of *E. coli* was based on gram staining, biochemical testing and culturing of each isolate on MacConkey agar and Eosin Methylene Blue agar (Figure 2), and PCR.

Biochemical tests used for the characterization of *E.coli* isolates from the water samples included: Methyl red (positive), Voges Proskauer’s (negative), Citrate utilization (negative), Indole (positive), Glucuronidase (positive), Nitrate reduction (positive), O-nitrophenyl-beta-D-galactopyranoside (ONPG) (positive), Lysine utilization (positive), Lactose (positive), Glucose (positive), Sucrose (the bacteria were variable for sucrose testing from 11%–89%), Sorbitol (positive), Catalase (positive) and Oxidase (negative). The positive control group was *E. coli* ATCC 8739. The identification of *E. coli* was confirmed by PCR amplification of the β-galactosidase gene, which gave a fragment of 243 bp.

### 3.3. Antibiotic Susceptibility

**Figure 3:**
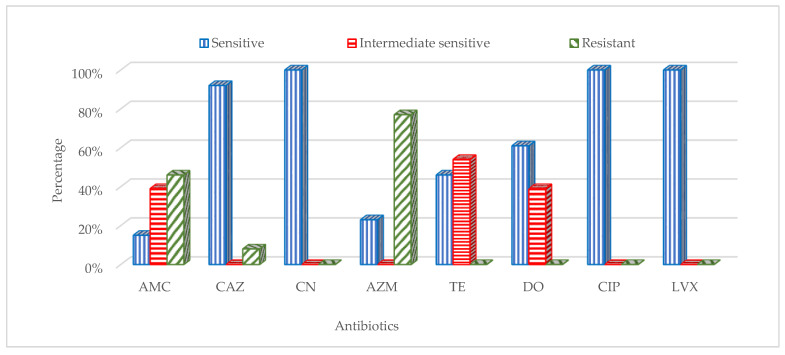
Resistance patterns of *Escherichia coli* isolates against the following antibiotics: doxycycline (DO, 30 mcg), ceftazidime (CAZ, 30 mcg), gentamicin (CN, 10 mcg), ciprofloxacin (CIP5, 5 mcg), azithromycin (AZM, 15 mcg), amoxicillin/clavulanic acid (AMC, 30 mcg), levofloxacin (LVX5, 5 mcg) and tetracycline (TE, 30 mcg).

The 13 isolates of *E. coli* were analysed for antibiotic susceptibility. The results of antimicrobial susceptibility testing showed that all *E. coli* isolates (13 of 13; 100%) were sensitive to gentamycin, ciprofloxacin and levofloxacin. The susceptibility of the isolates to ceftazidime, doxycycline, tetracycline, azithromycin and amoxicillin/clavulanic acid were 92%, 61%, 46%, 23% and 15%, respectively. Out of 13 *E. coli* isolates, 10 (77%), 6 (46%) and 1 (8%) were resistant to azithromycin, amoxicillin/clavulanic acid and ceftazidime, respectively (Figure 3).

## 4. Discussion

This study was conducted to investigate the presence of microbial contamination of coliform bacteria, particularly *E. coli*, in water used for drug compounding in community pharmacies in Jordan. Unfortunately, in Jordan there are no official guidelines or published resolutions establishing any standards for the quality of water or practices for drug reconstitution in local pharmacies. Additionally, there is a lack of information on the prevalence of pathogenic *E. coli* in water used for pharmaceutical compounding in community pharmacies in Jordan. To the best of our knowledge, we believe that this study is the first one conducted on screening water used in drug compounding in community pharmacies in Jordan.

The results of this study clearly indicate the presence of microbial contamination in the majority of water samples collected from community pharmacies (88%). The total microbial count was too numerous to count in most of the contaminated samples (58%) regardless of the source water, while only 12% of the water samples were free of microbial contamination. Such high levels of microbial growth may indicate accumulation of contaminations, mainly in the droppers themselves. However, the presence of contamination in the main water sources has not been tested, and therefore cannot be ruled out. A similar study was conducted in Brazil [23], where there are national regulations ensuring the quality of water used for drug compounding with quality control testing every month. That study included testing for coliform bacteria and *E. coli* in 744 samples from 30 pharmacies collected over a 4 year period and showed lack of evidence of coliform bacteria in all of the tested samples. Unfortunately, no other similar studies could be found in the literature. Nonetheless, the results of that study clearly indicate the potential effect of implementing regulations to ensure the quality of water used in reconstitution of medications, especially if such activities are practiced in hospitals or in reconstituting drugs for parenteral use.

In our study, most of the water samples from droppers filled with bottled water were contaminated with coliform bacteria. The chance of isolating coliform bacteria from bottled water was once tested in Tanzania, with 3.6% of the 131 bottled water samples testing positive for coliform bacteria [24]. None of the visited community pharmacies in our study had procedures for decontamination or instruments for sterilizing water droppers. Accordingly, and to overcome the chance of using contaminated bottled water, we recommend discarding used water droppers, or alternatively, using disposable bottled water from companies with known quality control status.

This study has shown that coliform bacteria were present in all water droppers sampled, regardless of the source of water. This may be directly related to the hygiene practices of the workers in the pharmacies rather than the level of contamination of the water source. Alternatively, the continuous filling of the same droppers without periodic decontamination or sterilization of the droppers may have resulted in accumulation of bacterial contaminants over time.

Due to the small size of sampled water from droppers filled with tap water, no conclusions could explain the lack of isolating coliform bacteria from such droppers. Nonetheless, the bacteriological quality of the tap water could be explained by the boiling step or the effectiveness of disinfection processes used for treating water before distribution.

In this study, 26% of the water samples collected from community pharmacies had *E. coli*, which poses a threat to the quality of compounded pharmaceutical products. The possible existence of coliform bacteria in treated drinking water may indicate a failure of treatment. The presence of antibiotic-resistant *E. coli* in water intended for drug compounding is a serious health risk to all consumers and particularly to immunocompromised individuals [25]. The risk of acquiring infections with such multidrug-resistant strains is further complicated by the fact that, in Jordan, several other sources of such strains can be obtained from surface water, drinking water and even from clinics [26,27,28].

The circulation of multidrug-resistant bacteria can be supported by the fact that the currently isolated *E. coli* were sensitive to gentamycin, ciprofloxacin and levofloxacin, while other isolates were resistant to azithromycin, amoxicillin/clavulanic acid and ceftazidime. This pattern is reflected in local clinical studies from environmental samples collected from hospitals and homes [28], as well as from patients in Jordan [29], which exhibited similar patterns of multidrug-resistant *E. coli*. The identification of similar resistance patterns in *E. coli* from these sites and from the sites sampled in this study might suggest the contribution of sub-optimal practices in local pharmacies in the dissemination of multidrug-resistant bacteria to human patients.

## 5. Conclusions

This study provides basic information about the presence of *E. coli* in water used for reconstitution of drugs in Jordanian community pharmacies. The current isolation of multidrug-resistant bacteria in most sampled water used for drug compounding is an alarming situation that needs special attention by pharmacists and competent authorities. Stricter measures should be implemented by the concerned regulatory authorities, such as the Jordan Food and Drug Administration and the Pharmacists Syndicate, to ensure high-quality compounding practices in the pharmacies and/or better conformation to the regulations of international pharmacopoeias. Regular inspection of water quality is expected to reduce the risk of microbial contamination in compounded products, particularly the multidrug-resistant strains of *E. coli* and other index microorganisms.

## Figures and Tables

**Table 1:** The targeted gene, primer sequence and product size.

Primer Name	Primer Sequence	Target Gene	Product Size	Reference
lacZ3	F: 5′ TTGAAAATGGTCTGCTGCTG 3′ R: 5′ TATTGGCTTCATCCACCACA 3′	β-galactosidase	243bp	[21]

**Table 2:** Total microbial and total coliform counts found in water samples used for reconstitution of drugs in Jordanian community pharmacies.

Total Coliform Count (CFU/100 mL)	Total *Escherichia coli* Count (CFU/100 mL)	Number of Samples (%)
TNTC	TNTC	1 (2%)
TNTC	1–10	8 (16%)
5–15	1–8	4 (8%)
3–30	Negative	11 (22%)
TNTC	Negative	20 (40%)
Negative	Negative	6 (12%)
**Total**		**50 (100%)**

TNTC: too numerous to count.

**Table 3:** Summary statistics on source of water in droppers used for pharmaceutical compounding in Jordanian community pharmacies. The table shows number of samples contaminated with *Escherichia coli*, mean (%) and standard error (SE). Statistical significance between source groups was inferred using Z-score and the *p*-values between the groups was reported.

Group Number	Water Source	Number of Samples (%)	Number (and %) of Samples Containing *Escherichia coli*	Percentage of Contamination (mean%; ±se)	Z-Score	*p*-Values
1	Cooler water	31 (62%)	8 (16%)	8/31 (25.8%; 7.9)	Between groups 1 & 2: −0.371406	0.355167
2	Bottled water	10 (20%)	2 (4%)	2/10 (20%; 12.7)	Between groups 2 & 3: −0.823688	0.205058
3	Boiled tap water	8 (16%)	3 (6%)	3/8 (37.5%; 17.1)	Between groups 3 & 1: 0.655681	0.256015
4	Tap water	1 (2%)	0 (0%)	0		
**Total (%)**		50 (100%)	13 (26%)			

## Data Availability

Not applicable.
